# Warming accelerated phosphorus release from the sediment of Lake Chaohu during the decomposition of algal residues: A simulative study

**DOI:** 10.1371/journal.pone.0314534

**Published:** 2025-01-15

**Authors:** Jiayu Hu, Qiong Zhao, Ping Zeng, Qiang Tang, Qingye Sun, Hongbin Yin

**Affiliations:** 1 School of Resources and Environmental Engineering, Anhui University, Hefei, China; 2 State Key Laboratory of Lake Science and Environment, Nanjing Institute of Geography and Limnology, Chinese Academy of Sciences, Nanjing, China; University of Kalyani, INDIA

## Abstract

Algal decomposition plays an important role in affecting phosphorus (P) release from sediments in eutrophic lakes under global warming. Yet how rising air temperature affect endogenous P release from sediments during the algal decomposition is poorly understood. In this study, effect of increasing air temperature on endogenous P release was investigated. A 22-day laboratory warming simulation experiment was conducted, with the overlying water and sediments collected from Lake Chaohu incubated in microcosms at three temperatures (21, 28 and 37°C). Dynamics of P fractions and related physiochemical properties in water and sediments were measured, and P release rate from sediments was calculated. Rising air temperature significantly reduced redox potential, but elevated pH, dissolved organic carbon (C) and alkaline phosphatase activity in water. For the average value during incubation, rising temperature significantly elevated P release rate and soluble reactive P by 3 times in overlying water, and greatly reduced total organic P (by 19.0%) in sediments, while did not affect total inorganic P in sediments. The NH_4_Cl-Po and NaHCO_3_-Po concentrations in sediments showed the greatest decrease (accounting for 97.6% of total decrease) during the experiment. Dynamics of P release rate, soluble reactive P, dissolved organic C in water and organic P, total organic C in sediments during incubation were also differed among different temperatures. The P release rate was significantly and negatively correlated with dissolved organic C and redox potential at all temperatures, negatively correlated with sediment inorganic P at 21°C, while negatively correlated with sediment organic P at 37°C. The results revealed that rising temperature strongly stimulated endogenous P release from sediments during the decay of algal residues, which was mainly due to the acceleration of organic P mineralization Warming-induced changes in the amount and dynamics of dissolved organic C played the dominant role in accelerating P release from sediments.

## Introduction

In recent years, global warming is aggravated and is causing dramatic environmental change global air temperature is predicted to rise by 1.8–4.0°C by the end of the century [1]. Warming can deeply influence the structure and function of eutrophic shallow lakes [2]. Eutrophication caused by excess anthropogenic nutrient input has been one of the most serious problems in lakes worldwide, and which is characterized by harmful algal blooms [3]. Recently, exogenous nutrient input has been effectively controlled in many eutrophic lakes, yet the water eutrophication and algal blooms would be continued for a long time due to endogenous nutrient release from nutrient-rich sediments and decay of algal residues. For example, for Lake Chaohu in China, external phosphorus (P) loads was 1.3 g P m^−2^ year^−1^ from 2014 to 2018, while the diffusive flux of soluble reactive P from sediments reached to 2.76 mg m^−2^⋅d^−1^ [4, 5]. P is one of the main nutrient elements involved in lake eutrophication, and its internal cycle within the lake can be greatly affected by warming [6]. Although endogenous P release from lakes sediments was extensively studied, its relationship with decay of algal residues was poorly known [7]. Therefore, to better understand internal P transformations in eutrophic lakes under the background of global warming, it is necessary to reveal the mechanisms and dynamics of endogenous P release during the decay of algal residues under rising air temperature.

Rising temperature was considered to accelerate the endogenous P release from sediments in eutrophic lakes through multiple pathways [8]. Firstly, temperature increase can greatly affect redox potential (Eh), pH, and dissolved organic carbon (C) and thus accelerate the desorption and dissolution of recalcitrant inorganic P in sediments. With increasing temperature, Eh in water reduced, due to decreased oxygen dissolution and increased oxygen consumption by microorganisms and planktons [9]. The reduction in Eh can accelerate the reduction of Fe(III) to Fe(II), and thus promote the release of Fe-bound P [10]. In a simulative experiment with the temperature rising from 8°C–22°C, reduction in NaOH extractable Al and Fe bound P accounted for 79.3% of total reduction in sediment P in Hongfeng Lake [11]. pH was a predominant factor controlling P release from sediments [12]. The rate of P release decreased as pH increased from 2 to 6 in Taihu Lake, while it increased as pH increased from 8 to 12. It is suggested that high pH promoted the release of NaOH-P, and low pH promoted the release of HCl-P, and there was no P release occurring in the neutral condition [13], because more phosphates are precipitated with Fe/Al or Ca compounds and adsorbed on Fe/Al oxides [14]. pH can also greatly affect phosphatase activities and thus mineralization of organic P. Sediment organic P mineralization was found to be significantly higher at pH around neutral (approximately 7.4) than at higher pH under both aerobic and anerobic conditions [15]. Variation in dissolved organic C was considered an important way by which rising temperature mobilize recalcitrant inorganic P in sediments [16]. Rising temperature can accelerate the decomposition of organic matter and thus elevate the concentration of dissolved organic C, which strongly competes with phosphate for adsorption sites [17]. Concurrent increases in soluble reactive P and dissolved organic C were observed in sediments with increasing temperature [18].

Rising temperature can promote microbial biomass and phosphatase activities and thus accelerate the mineralization of organic P in sediments [19]. In an incubation experiment with the temperature rising from 5°C to 25°C, the decrease in sediment P with rising temperate was mainly in the form of organic P [20]. In most of lakes, inorganic P dominate sediment P, organic P accounts for only 12–60% of total P [21]. Previous studies on the endogenous P release from sediments and its responses to rising temperature mainly focused on inorganic P fractions. However, considering the strong responses of microorganisms to rising temperature, organic P transformations could play an unneglected role in warming-induced P release from sediments. Rising temperature can also affect P release from sediments in lakes indirectly by influencing the uptake of P by algal growth and release of P from decay of algal residues, yet which is rarely investigated [22].

Algal decomposition is a key process of internal P cycling in eutrophic lakes, which can not only directly release phosphate to water, but also affect P release from sediments [7, 23]. Yet so far, there is limited research on how the algal decomposition affects P release from sediments. Being concurrent with the release of phosphate, rapid algal decomposition also release abundant of dissolved organic C, which can influence transformations of both organic and inorganic P in sediments [24, 25]. Firstly, dissolved organic C had the “priming effect” on the mineralization of organic C and P in sediments. That is, the input of dissolved organic C improved the microbial biomass and activities, and thus accelerated the mineralization of more stable organic matter originally existing in sediments [26, 27]. In addition, decomposition of organic matter consumes the oxygen and thus reduce the Eh, and consequently accelerated the release of Fe-bound P from sediments [28, 29]. The increase in dissolved organic C can also accelerate the release of P adsorbed on minerals in sediments due to its competition with phosphate for adsorption sites [30]. Decomposition of algal residues also changed water pH, but the results varied greatly under oxic and anoxic conditions [31]. Decomposition of algal residues released large amounts of CO_2_ and organic acids into the water, and had the potential to reduce pH under oxic conditions [32]. In contrast, under anoxic conditions, reduction of Mn(IV), Fe(III), and sulfate may increase the pH and thus counteract the effect of CO_2_ formation, or even elevate water pH [33]. It has been reported that there are positive feedbacks between algal blooms, climate warming and lake eutrophication. That is, warming promotes algal blooms, and then rapid growth and decay of algae aggravate eutrophication and emission of greenhouse gases [22]. Therefore, algal decomposition would play a more important role in affecting P release from sediments in eutrophic lakes under rising temperature, and experimental studies are needed to examine this hypothesis.

The objective of this study was to reveal how rising temperature affect endogenous P release from sediments during the algal decomposition in eutrophic lakes. To achieve this goal, a laboratory warming simulation experiment was conducted. Water and surface sediments collected from a eutrophic shallow lake were incubated with the algal residues at three temperatures for 22 days. Temporal dynamics of P and related physiochemical properties in water and sediments were examined. We hypothesized that: (1) endogenous P release from sediments would increase with rising temperature; (2) dynamics of P released from sediments would be correlated with the algal decomposition, and which would be different at the three temperatures, since warming will accelerate the decomposition of algal residues; (3) release of organic P forms from sediments would increase with rising temperature, because both warming and algal decomposition will accelerate organic P mineralization in sediments. It is hoped that the results will help to clarify the correlation between internal P cycle within eutrophic lakes and global warming, and provide guidance for the control of endogenous P release.

## Materials and methods

### Site description

Lake Chaohu is the fifth largest freshwater lakes in China, which locates in Anhui Province in southeastern China (31°25′–31°30′N, 117°16′–117°51′E). The lake belongs to a subtropical monsoon climate zone, with multiyear mean temperature of 16.1°C and average annual precipitation of 1003 mm [2, 34]. The lake covers an area of approximately 770 km^2^ with an average depth of 3 m. There are thirty-three rivers flow into Lake Chaohu, which brought a large amount of nutrients and pollutants from industry and agriculture to the lake, and has resulted in severe eutrophication and algal blooms in the past two decades [35]. Eutrophication of the lake was most serious in western past, where is surround by agricultural areas. Correspondingly, nitrogen (N) and P concentrations in the water and sediments of Lake Chaohu were highest in the northwest bay area, and then gradually decrease from the center to eastern area [36]. In recent years, external input of nutrients and pollutants to Lake Chaohu was effectively controlled. However, N and P in the water remain at a relatively high level in the lake, especially in the western part, due to the endogenous release of N and P from sediments [37].

### Sample collection and experiment design

In April 2023, the water and sediment used for the laboratory experiment were sampled from the northwest bay of Lake Chaohu (31°70′N, 117°37′E) where nitrogen (N) and P concentrations were higher than other areas of the lake. For the water and sediment sampling, we set five sampling sites, which includes a sampling site at the center of the northwest bay of Lake Chaohu, and four sampling sites were around the center of the northwest bay of Lake Chaohu. Samples of water and sediments from the five sites were homogenized into one sample for the laboratory experiment. A plexiglass hydrophore was used to collect the water (with the algae). Surface sediments (0~15 cm) were collected using a grab sampler. We measured temperature, pH, dissolved oxygen (DO), electrical conductivity (EC) and redox potential (Eh) in situ with a HACH HQ30D Portable meter. The filed sediments were divided into two parts, one part was freeze-dried for the measurement of initial biochemical properties. Another part was used for the laboratory warming experiments.

A 22-day laboratory simulation experiment with the algal, water and sediments incubated in microcosms at three temperatures was conducted, to simulate the impacts of warming on P dynamics in water and sediments of Lake Chaohu. The microcosms were placed in constant temperature incubators, and temperatures of the incubators were set to 21°C, 28°C and 37°C, respectively, which are corresponding to the air temperature of 20°C, 30°C and 40°C in the field. The incubation temperatures were calculated in light of the fitting formula of water and air temperatures in Lake Chaohu [38]. We set the maximum air temperature at 40°C for the reason that maximum temperature of Lake Chaohu has reached to 39°C in 2022 (from China National Meteorological Data Service).

In view of the fact that algae release algae-derived organic matter into the water when they die, which provides easily degradable substrate for methanogens and stimulates the production of CH_4_ [39], the actual temperature of the incubator monitored daily exceeds the set temperature. Polyvinyl chloride (PVC) tubes with an inner diameter of 76 mm and a height of 450 mm were used for the water and sediment incubation. A total of 54 tubes were used, to make sure three tubes were sampled every time for each treatment. Firstly, field sampled sediments were added to the PVC tube up to 10 cm thick. Then, the overlying water sample was gently added to the tube until it reached a height of 30 cm above the sediment (The initial concentration of chlorophyll a in water sample was 55.8 μg L^−1^). Then, the PVC tube is pre-incubated for three days at the room temperature. *Dolichospermum flos-aquae* and *Microcystis aeruginosa* were the dominant cyanobacteria in the lake, and their decomposition process lasted about 5–20 days [40, 41]. Therefore, to investigate the impacts of decomposition of algal residues on P release from sediments, all tubes were kept in dark for 22 days. Water and sediments were sampled on the 1st, 5th, 9th, 13th, 17th and 22nd days of the simulation experiment, and measured for biochemical properties.

### Laboratory analyses

Water samples collected during the simulation experiment were filtered through a 0.45 μm membrane, and measured for pH, Eh, EC, chlorophyll a (Chl-a), dissolved organic C (DOC), alkaline phosphatase activity (AlPase), total P, dissolved total P (TDP) and soluble reactive P (SRP). Water pH and EC were measured using a thunder magnetic pH meter. Eh were measured using an HACH HQ30D portable meter (HACH, USA). DOC was filtered by 0.45 μm glass fiber membrane and determined by TOC-L analyzer (Elementa vario, Germany). Chl-a was extracted by hot alcohol and determined by spectrophotometer [42]. AlPase activity was determined by the method of Tabatabai [43], using the p-nitrophenyl phosphate (pNPP) as substrate. Enzyme activity was expressed as the production of p-nitrophenol (pNP) from p-nitrobenzene disodium phosphate (pNPP) culture. Concentration of SRP in water was determined by molybdenum antimony colorimetric method [44]. Total P and TDP were determined bymolybdenum blue method after the water samples were digested by potassium persulfate.

Sediment samples collected during the simulation experiment were freeze-dried and sieved through a 0.15 mm diameter mesh, and measured for pH, organic C, AlPase activity, total P, inorganic P and P fractions. Sediment pH was measured using a glass electrode with the sediment/water ratio of 1/5 (*w*/*v*). Organic C was determined by loss-on-ignition method in a muffle furnace at 550°C for 6 h [45]. AlPase activity in sediments was measures using the same method as in water samples. Total P and inorganic P were extracted by the standards, measurements and testing (SMT) method, and measured by molybdenum antimony colorimetric method [46].

**Fig 1:**
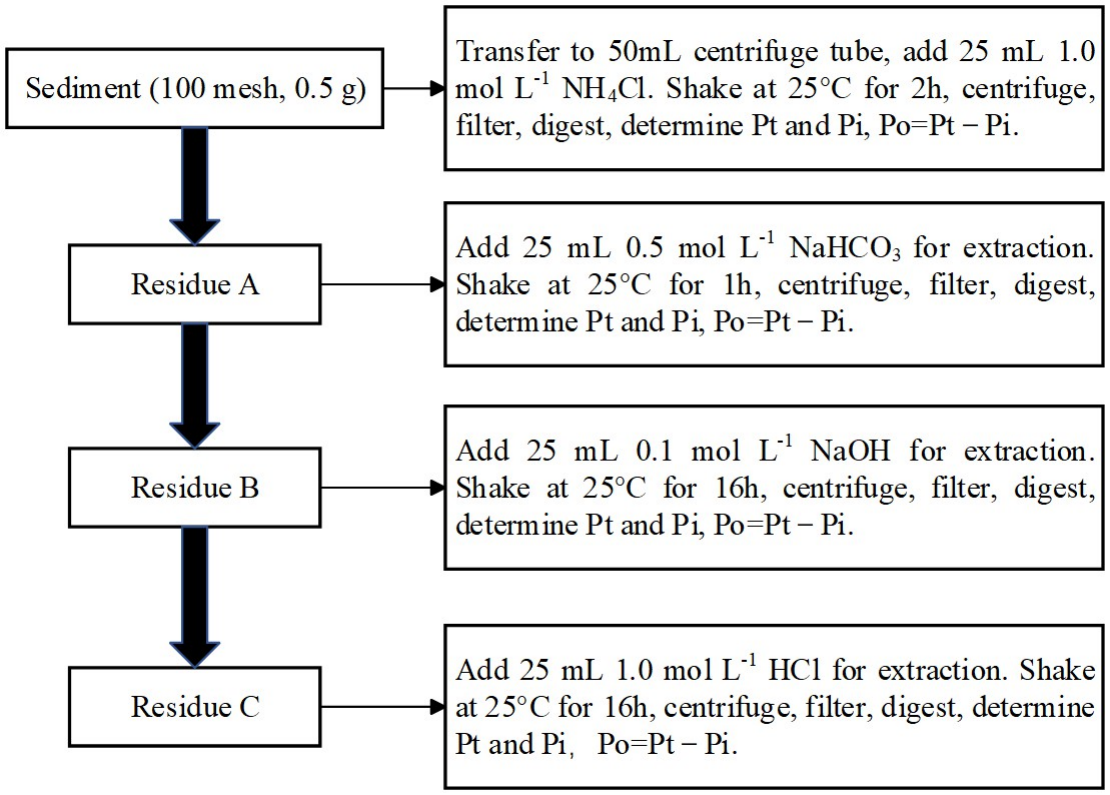
Flow chart of sediment P fractionation procedure.

Sediment P was separated into operationally defined organic and inorganic pools using a modified Hedley’s continuous extraction method [47, 48]. Chemical reagents with increasing dissolution strength were used to successively remove more recalcitrant forms of P from the sediments. Briefly, 0.5 g of sediment was successively extracted with 1.0 mol L^−1^ NH_4_Cl (pH = 7.0), 0.5 mol L^−1^ NaHCO_3_, 0.1 mol L^−1^ NaOH, 1 mol L^−1^ HCl. Inorganic P concentrations in all the extracts (NH_4_Cl-Pi, NaHCO_3_-Pi, NaOH-Pi and HCl-Pi) were determined by the molybdenum antimony colorimetric method. Total P concentrations in all the extracts were determined after acid persulfate digestion (autoclaving for 30 min at a pressure of 1.05 kg cm^−1^ and 121°C) [49]. Organic P concentrations in the extracts (NH_4_Cl-Po, NaHCO_3_-Po, NaOH-Po and HCl-Po) were calculated as the difference between total P and inorganic P (Fig 1).

In the sequential extraction procedure, extractants remove soil P with different bioavailability. NH_4_Cl and NaHCO_3_ solutions extract labile P fractions, which mainly include phosphate in soil solution, inorganic P weakly adsorbed on the surface of crystalline compounds, carbonate, and easily mineralized organic P [50]. NaOH solution extracted partially dissolved Fe-Al phosphates and inorganic P strongly chemisorbed to surfaces of amorphous and some crystalline Al and Fe, and organic P associated with humic and fulvic acids [51]. HCl solution extract strongly fixed P fractions that are mainly primary mineral P compounds tightly bound with Ca [47].

Sediment P release rate (mg m^−2^ d^−1^) was used to evaluate the rate of P transferred from the sediment to overlying water, according to the following equation [52]:

$$\text{P}\;\text{release}\;\text{rate}=\frac{C_{after}-C_{before}\times V}{S\times T}$$

where C_after_ and C_before_ is the SRP concentration in the overlying water after and before the simulation experiment (mg L^−1^); V is the volume of overlying water (mL); S is the cross-sectional area of the simulation pipe (m^2^); and T is the interval between two sampling dates (days).

### Statistical analyses

Two-way analysis of variance (ANOVA) with repeated measurements was conducted to test for the main effects of temperature (T) and incubation time (D) and their interactions (T × D) on water and sediment variables. One-way ANOVA Tukey’s HSD multiple comparison was performed to compare the differences among three temperatures in each sampling date for all variables. The average values of physiochemical properties throughout the simulation experiment were also calculated and compared among the three temperatures using one-way ANOVA. Levene’s test was used to test for homogeneity of error variances prior to ANOVA, and non-parametric test (Kruskal-Wallis test) was used when the data did not meet ANOVA assumptions. Statistical significance was set at *p*<0.05.

Pearson’s correlation analyses were conducted to analyze the relationship between P release rate and variables in water and sediments at each temperature separately, and between sediment P fractions and physiochemical properties to reveal the key factors controlling P release rate. To further estimate and better visualize how water and sediment biochemical properties influence sediment P fractions, redundancy analysis (RDA) was used to estimate the relationships between sediment P fractions and water and sediment variables across all temperatures. RDA with scaling II was conducted, with biochemical properties as explanatory variables, and P fractions without transformation as response variables. Monte Carlo permutation test (999 unrestricted permutations) and multicollinearity test were used for pre-selection of explanatory variables. The ANOVA and correlation analyses were performed using SPSS 26.0 (IBM, Chicago, USA), and RDA was performed using CANOCO 5.0 software. All figures were drawn with Origin 2021.

## Results

### Physicochemical properties in the overlying water and sediments

**Fig 2:**
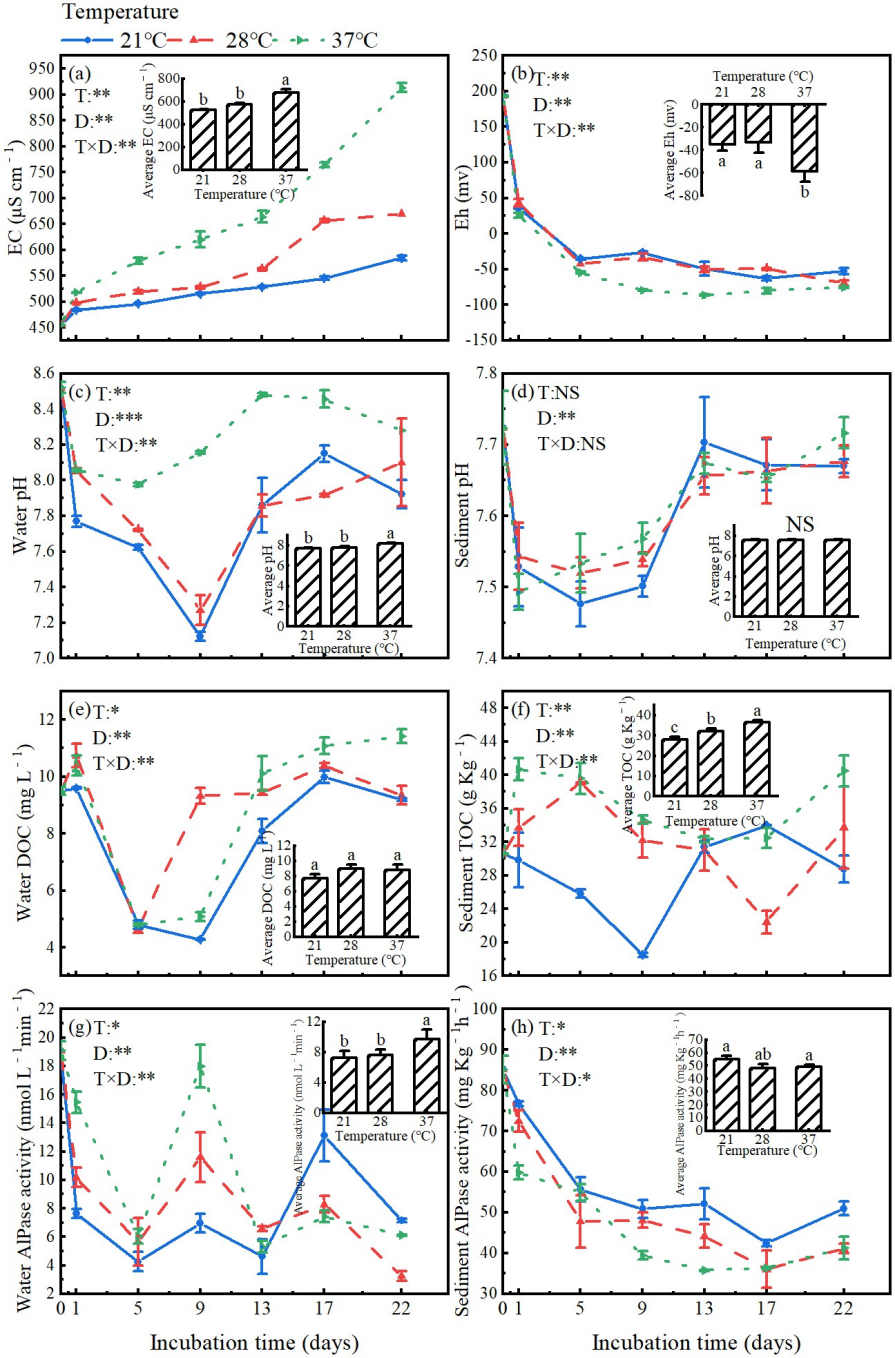
Changes in physicochemical properties: (a) EC, (b) Eh, (c) water pH, (d) sediment pH, (e) water DOC, (f) sediment TOC, (g) water AlPase activity and (h) sediment AlPase activity.

ANOVA analyses showed that all measured physicochemical properties in the overlying water and sediments changed significantly during the incubation period, and were significantly affected by incubation temperature, except that sediment pH were not affected (Fig 2, S1 Table). Furthermore, there were significant interactions between temperature and incubation time on these variables except for sediment pH (Fig 2).

Average values of these variable during the incubation period were compared among the three incubation temperatures (Fig 2). In the overlying water, average values of pH, EC, and AlPase activity all significantly increased (by 6.5%, 28.7%, and 33.3%, respectively), while Eh significantly reduced (by 66.9%), and DOC did not change, with increasing temperature. Furthermore, the differences of these variables were significant between 37°C and the two lower temperatures. In the sediment, the average values of total organic C significantly increased (by 30.9%), while AlPase activity decreased (by 11.9%) from the lowest to highest temperature. Average value of pH in sediments did not change with rising temperature.

In the overlying water, EC increased continuously, while Eh reduced continuously throughout the entire incubation period at all temperatures, both to a larger extent at 37°C than at 21°C and 28°C (Fig 2a and 2b). Eh was negative at all temperatures, showing a strong reduction state. Water pH changed during incubation in the similar patterns at all temperatures, declined sharply at first and then increased gradually (since the 5th or 9th day), generating a net decrease (by 0.24~0.60 unit) in pH at the end of the incubation relative to the initial value. Besides, the decline in water pH at the early incubation stage was much smaller at 37°C than at 21°C and 28°C (Fig 2c). At all temperatures, pH in the sediments declined rapidly at first and then increased, generating the unchanged pH at the end of the experiment relative to the initial value (Fig 2d).

Concentration of DOC in the overlying water also changed significantly during incubation in the same pattern at all temperatures, declined at first and then increased (since the 5th or 9th day), generating almost unchanged DOC at the end of the incubation relative to the initial value, except that it elevated by 20.3% at the highest temperature (Fig 2e). During the incubation period, total organic C in the sediment decreased at the early stage, and then increased at all temperatures, but the lowest value occurred on different date at three temperatures (Fig 2f). Variation in AlPase activity during the incubation in overlaying water exhibited a single-peak shape in all temperatures, with the peak values occurring on different date at the three temperatures (Fig 2g). However, the AlPase activity in sediments reduced gradually during the entire incubation period, to a greater extent at 37°C and 28°C than at 21°C (Fig 2h).

### P fractions in the overlying water and P release rate from sediments

**Fig 3:**
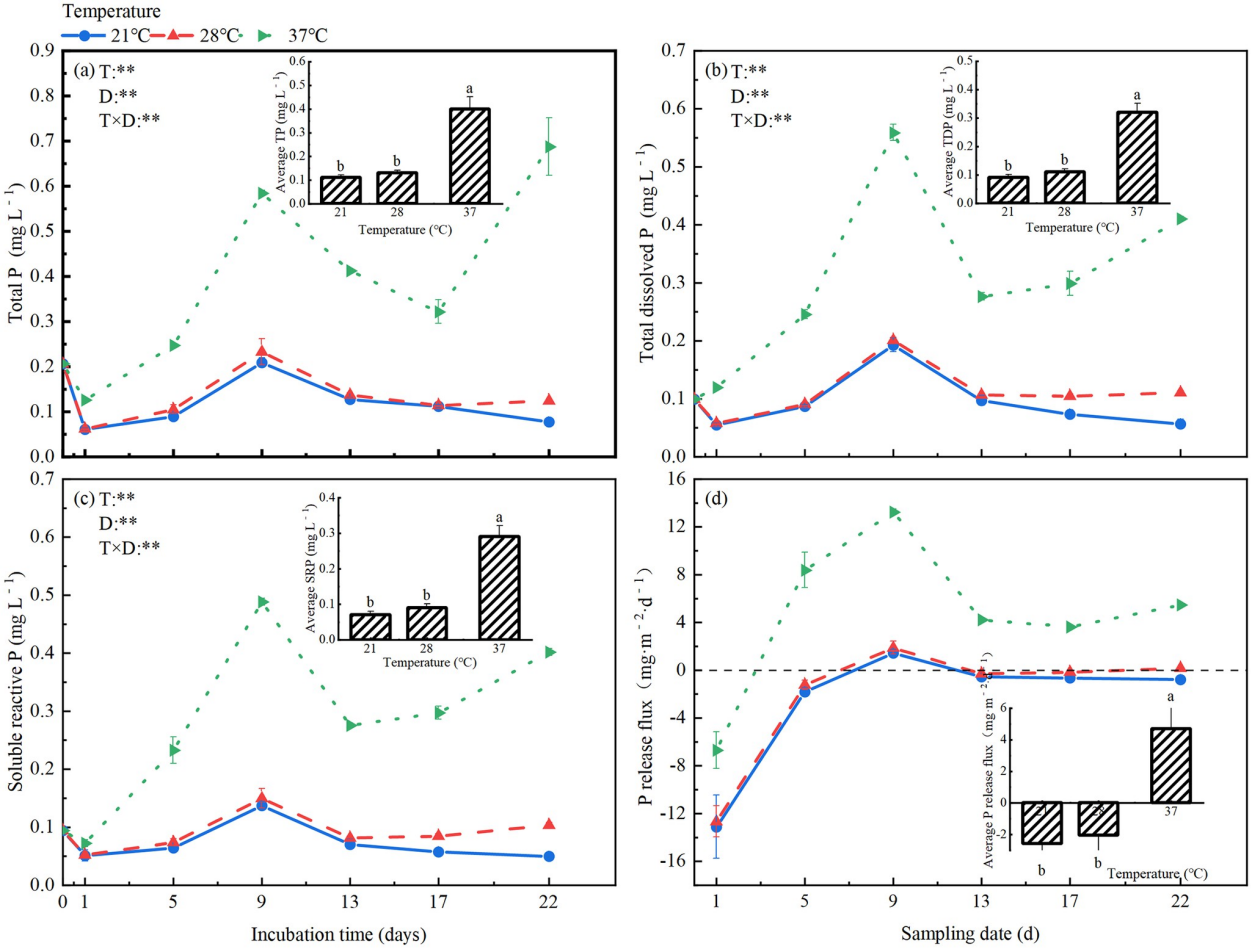
Water P fractions and sediment P release rate at different temperatures: (a) total P, (b) total dissolved P, (c) soluble reactive P of water and (d) sediment P release rate.

ANOVA analyses showed that total P, DTP and SRP in the overlying water changed significantly during incubation, and were significantly affected by incubation temperature. Furthermore, there were significant interactions between incubation time and temperature on these variables (Fig 3a–3c). Average values of total P, DTP and SRP during the incubation period were compared among the three temperatures, all of them were greatly higher at 37°C than at 21°C and 28°C (2. 91~4.14 times), but did not differ between 21°C and 28°C (Fig 3a–3c).

During the incubation, total P in the overlying water changed in different patterns at different temperatures (Fig 3a). It changed in a unimodal pattern with an obvious peak (3.5–3.8 times of the lowest value) on the 9th day at 21°C and 28°C, generating an unchanged value at the end of the incubation relative to the initial value. In contrast, total P fluctuated dramatically at 37°C, it similarly reached a peak on the 9th day as at 21°C and 28°C, but rose again sharply since 17th day (from 0.13 mg L^−1^ to 0.69 mg L^−1^), resulting in a greatly higher value (3.29 times) at the end of incubation relative to the initial value (Fig 3a). DTP and SRP changed during incubation in the same pattern as total P at the corresponding temperatures. At the end of the incubation at 37°C, DTP and SRP were 4.10 and 4.44 times of their initial values (Fig 3b and 3c). Sediment P release rate increased at first, then decreased and kept constant during incubation at all temperatures, but the increase at the early stage was much greater at 37°C than at 21°C and 28°C (Fig 3d). Consequently, P release rate was significantly higher at 37°C than at 21°C and 28°C throughout the incubation period, but did not differ between 21°C and 28°C.

### P fractions in the sediment

**Fig 4:**
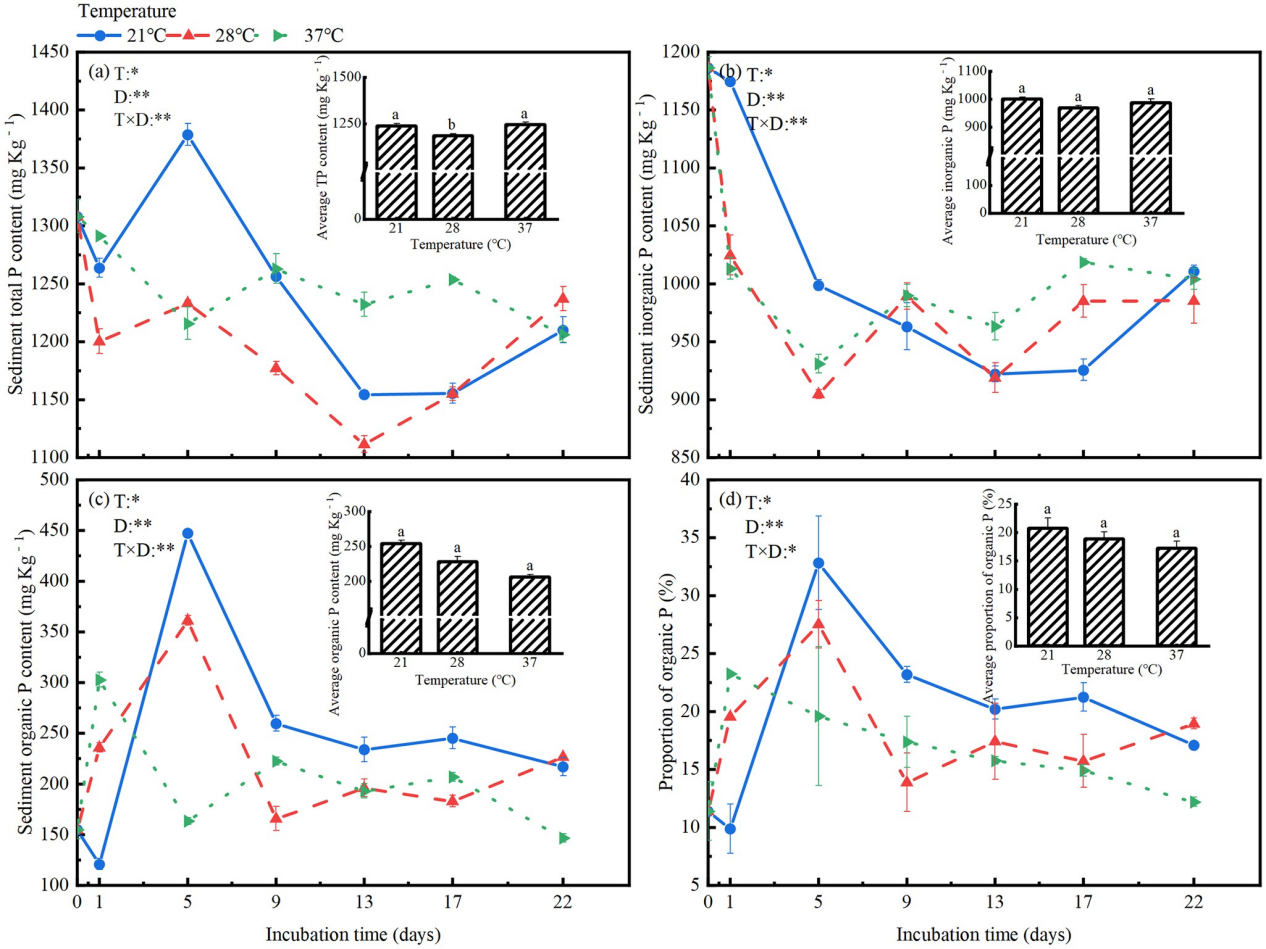
Changes in total, organic and inorganic P fractions at different temperatures: (a) total P, (b) total organic P, (c) total inorganic P and (d) proportion of organic P.

ANOVA analyses showed that the total P, inorganic P, organic P and proportion of organic P in sediments changed significantly during incubation, and were significantly affected by incubation temperature. There were significant interactions between incubation time and temperature on these variables (Fig 4). Average values of these variable during the incubation period were compared among the three incubation temperatures (Fig 4). In the sediment, average values of organic P (by 19.0%) and proportion of organic P (by 17.2%) reduced gradually with rising temperature, while no significant differences for total P and inorganic P among three temperatures were observed (Fig 4).

During the incubation, total P concentration declined in fluctuation, and inorganic P declined continuously at all temperatures (Fig 4a and 4b). At the end of the incubation, total P and inorganic P were about 94.6% and 85.2% of the corresponding initial value. Organic P fluctuated throughout the incubation period at all temperatures, with a great increase on the first day at 37°C, and on the 5th day at 21°C and 28°C. At the end of the incubation, organic P was 5.3% lower than the initial value at 37°C, while was 40.2% and 46.4% higher than the initial value at 21°C and 28°C (Fig 4c). Organic P accounted for 9.9%~32.8% of total P, and this proportion fluctuated in the same pattern as the organic P concentration (Fig 4d).

**Fig 5:**
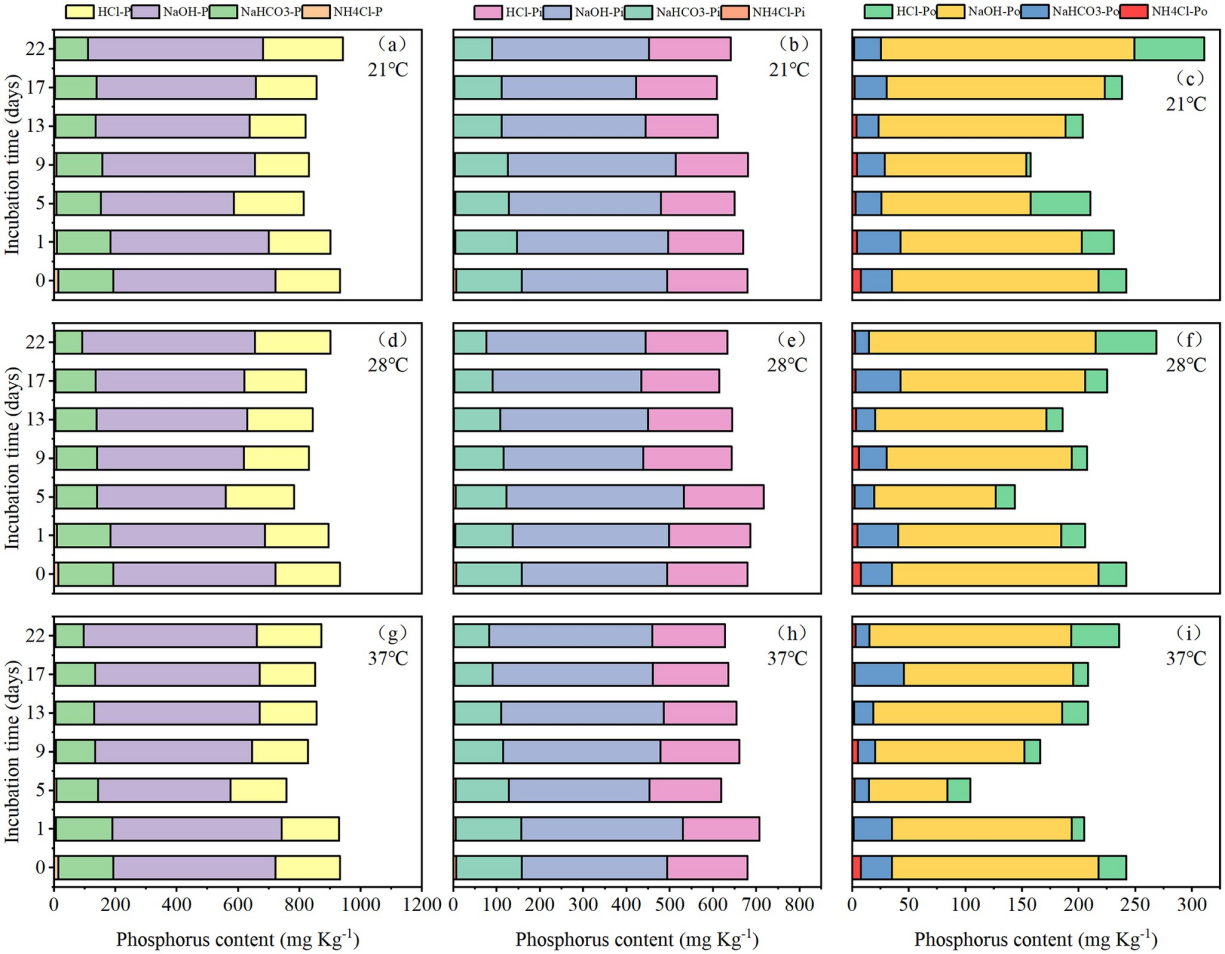
Changes in sediment P fractions at different temperatures.

Among the sequentially extracted P fractions, NaOH-Pt was the predominant P fractions, then the concentration decreased in the order of HCl-Pt > NaHCO_3_-Pt > NH_4_Cl-Pt. At the end of the incubation, total P in all extractants (except NH_4_Cl fraction) were lower at 28°C and 37°C compared to 21°C (Fig 5). Throughout the incubation period, NaHCO_3_-Pt and NH_4_Cl-Pt reduced continuously, while NaOH-Pt and HCl-Pt varied undulately.

Concentrations of inorganic fractions decreased in the order NaOH-Pi > HCl-Pi > NaHCO_3_-Pi > NH_4_Cl-Pi. After 22 days of incubation, NH_4_Cl-Pi, NaHCO_3_-Pi and HCl-Pi concentrations were significantly lower (by 10.7%~24.7%) at 28°C and 37°C than at 21°C. By contrast, NaOH-Pi concentration had slight changes (< 5.0%) at 28°C and 37°C compared to 21°C at the end of the incubation. Throughout the incubation period, the concentrations of NH_4_Cl-Pi and NaHCO_3_-Pi in sediments decreased continuously, while NaOH-Pi, and HCl-Pi concentrations fluctuated, resulting in 78.7%~83.9% and 41.7%~50.1% decreases in NH_4_Cl-Pi and NaHCO_3_-Pi, 8.0%~12.4% increases in NaOH-Pi, and unchanged in HCl-Pi at the end of the incubation relative to the initial values (Fig 5). For average values throughout the incubation period, there were no significant differences between three temperatures for all Pi fractions, except that HCl-Pi concentration was significantly (10.1%) higher at 28°C than at 37°C (S1 Fig).

Concentrations of Po fractions decreased in the order NaOH-Po > NaHCO_3_-Po and HCl-Po > NH_4_Cl-Po. At the end of the incubation, all organic P fraction significantly decreased with rising temperature with the lowest values at 37°C, except that NH_4_Cl-Po concentration significantly increased by 12.3% from 21°C to 28°C. Concentration of NH_4_Cl-Po, NaHCO_3_-Po, NaOH-Po and HCl-Po were 34.6%, 14.5%, 14.4%, and 37.3% lower at 37°C than at 21°C, respectively (S1 Fig). Throughout the incubation period, the concentrations of all Po fractions in sediments fluctuated, resulting in 56.9%~75.8% and 11.9%~57.8% decreases in NH_4_Cl-Po and NaHCO_3_-Po, 7.6%~18.6% and 65.1%~130.4% increases in NaOH-Po and HCl-Po at the end of the incubation relative to the initial values (Fig 5). For average values throughout the incubation period, NH_4_Cl-Po concentration was significantly (28.7%~44.6%) higher at 21°C and 28°C than at 37°C, NaOH-Po and HCl-Po concentration were significantly (16.8%, 59.5%) higher at 21°C than at 37°C (S1 Fig).

### Relationships between P release rate, sediment P and physiochemical properties

**Fig 6:**
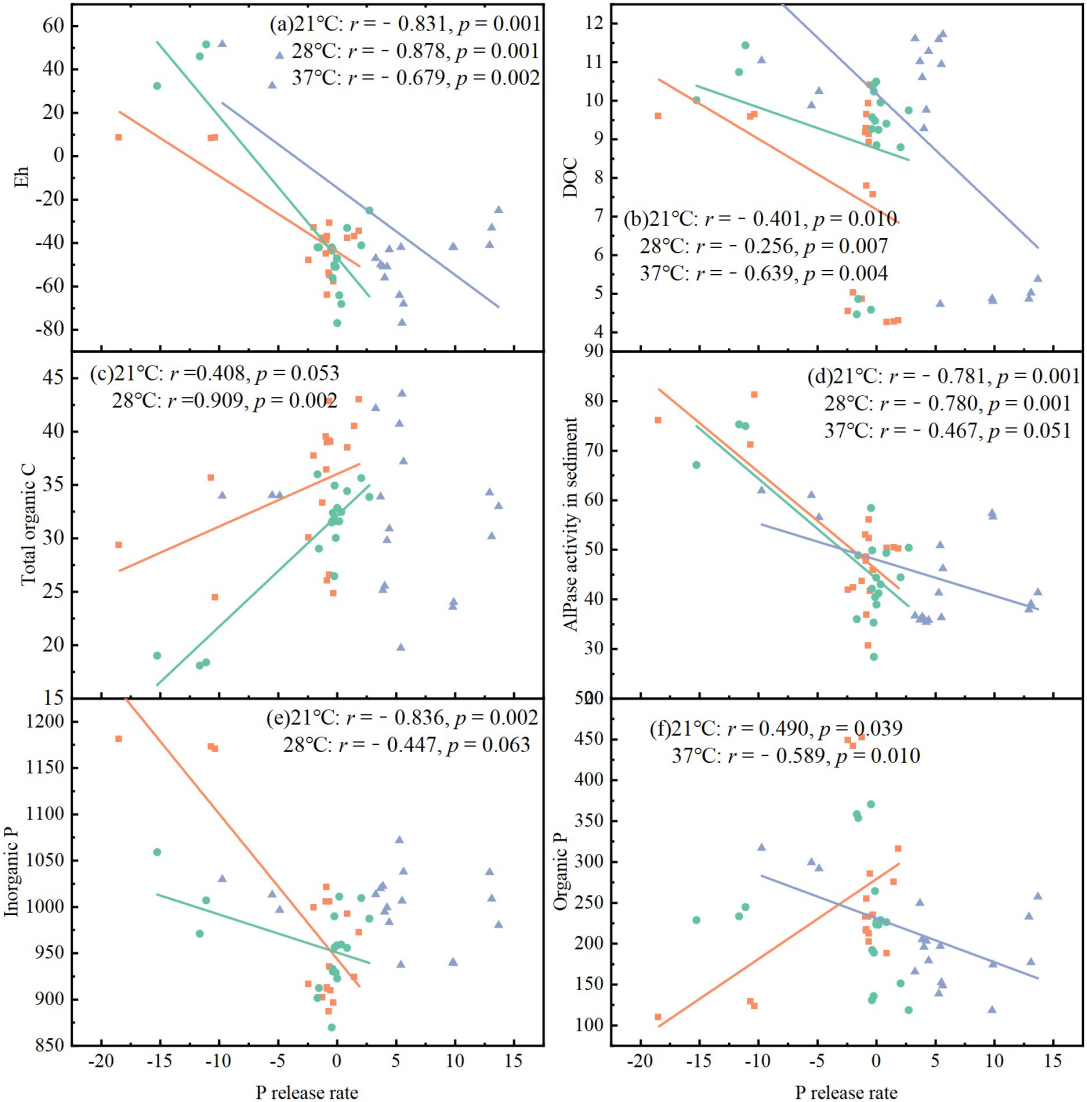
Relationships between P release rate and Eh (a), DOC (b) in water, and total organic C (c), AlPase activity (d), inorganic P (e), and organic P (f) in the sediment.

Correlations between P release rate and various water and sediment properties were analyzed at different temperatures (Fig 6, S3 Table). Eh in the water was significantly and positively correlated with P release rate at all the temperatures (*r* = −0.831, −0.878 and −0.679, *p* < 0.01, Fig 6a). DOC in the water was negatively correlated with P release rate at all temperatures, with a stronger correlation at the 37°C (*r* = −0.639, *p* = 0.004, Fig 6b) than at 21°C and 28 °C (*r* = −0.401 and −0.256, *p* < 0.05). pH in both water and sediments, and AlPase activity in the water did not correlate with P release rate at all temperatures (*p*>0.05, S3 Table). Total P, TDP and SRP in the water were positively correlated with P release rate at 37°C and 28°C (*r* = 0.564~0.825, *p* < 0.05, S3 Table), but not at 21°C (*p*>0.05). Correlations between sediment variables with P release rate highly depended on temperature. Sediment total organic C was positively correlated, and AlPase activity was negatively correlated with P release rate at 21°C and 28°C (*r* = 0.525~0.909, *p* < 0.05; *r* = −0.780, *p* < 0.01, Fig 6c and 6d), but not at 37°C (*p*>0.05). Inorganic P in the sediment was positively correlated with P release rate only at 21°C (*r* = −0.833, *p* < 0.01, Fig 6e). In contrast, organic P positively correlated with P release rate at 21°C (*r* = 0.486, *p* < 0.05, Fig 6f), while negatively with it at 37°C (*r* = −0.555, *p* < 0.05), but not correlated with it at 28°C (*r* = −0.176, *p*>0.05).

Correlations between sediment P fractions and physiochemical properties were analyzed at each temperate separately (S2 Table, S2 Fig). Almost at all temperatures, NH_4_Cl-Pi and NaHCO_3_-Pi were both significantly positively or negatively correlated with EC, Eh, sediment pH and AlPase activity (*r* = −0.832‒0.680, *p* < 0.01). NaHCO_3_-Pi was also strongly and negatively correlated with water pH and DOC (*r* = −0.272 and −0.291, *p* < 0.05). HCl-Pi was significantly and positively correlated with the water AlPase activity (*r* = 0.286, *p* < 0.05). NH_4_Cl-Po was significantly negatively correlated with the water pH (*r* = −0.292, *p* < 0.05). NaHCO_3_-Po was also significantly and positively correlated with the water Eh, DOC and AlPase activity (*r* = 0.358–0.454, *p* < 0.01), but was negatively related with the Ec (*r* = −0.304, *p* < 0.05). NaOH-Po was significantly positively related with sediment pH, organic C, EC and DOC (*r* = 0.296–0.446, *p* < 0.05). HCl-Po was significantly negatively correlated with the water AlPase activity (*r* = −0.520, *p* < 0.01).

**Fig 7:**
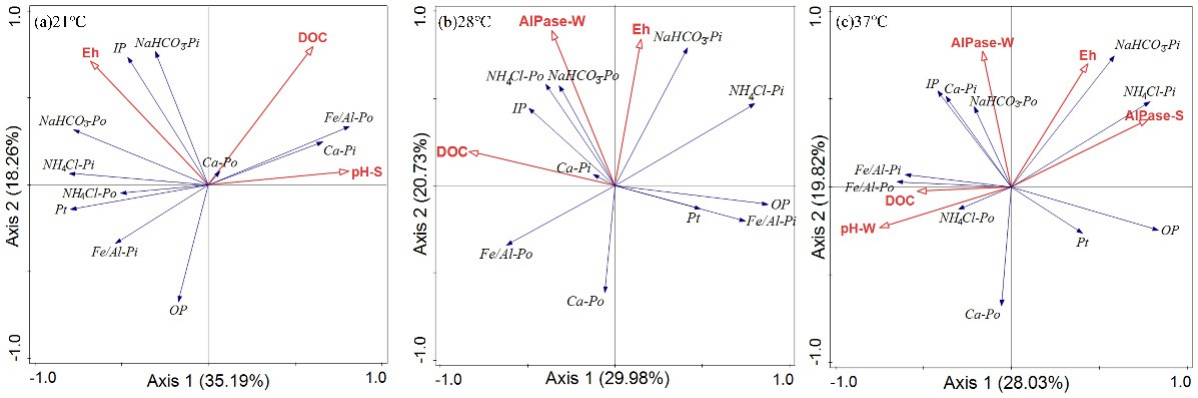
Ordination diagram from constrained redundancy analysis (RDA) of the relationship between the P fractions and those of water and sediment biochemical properties in three temperatures.

RDA results showed that biochemical properties explained 61.6%, 54.2% and 70.3% of the variations in sediment total P and P fractions at different temperatures, respectively. At 21°C, RDA1 and RDA2 explained 35.19% and 18.26% of the variations in total P and P fractions, respectively. The Eh, DOC and sediment pH were factors significantly contributed to the variations in total P and P fractions, and which explained 18.9%, 16.8%, and 25.9% of the observed total variances in total P and P fractions. At 28°C, RDA1 and RDA2 explained 29.98% and 20.73% of the variations in total P and P fractions, respectively. The DOC, Eh and water AlPase activity were factors significantly contributed to the total P and P fractions, and which explained 22.6%, 19.9% and 11.7% of the observed total variances in total P and P fractions. At 37°C, RDA1 and RDA2 explained 28.03% and 19.82% of the variances in total P and P fractions, respectively. The sediment AlPase activity, Eh, DOC, water AlPase activity and water pH were factors significantly contributed to the total P and P fractions, and which explained 22.3%, 17.8%, 13.9%, 9.4% and 6.8% of the observed total variances in total P and P forms (Fig 7).

## Discussion

### Warming-induced changes in DOC stimulated P release from sediments

Being consistent with our first two hypotheses, warming greatly elevated P release from sediments and changed its temporal dynamics during the algal decomposition (Figs 3 and 4). Warming and the algal decomposition can stimulate P release from sediments synergistically, via changing Eh, pH and dynamics of DOC. In the present study, the dynamics of DOC could play the predominant role in stimulating P release from sediments under rising temperature. This was suggested by the significant and negative correlations between DOC and P release rate and sediment P fractions, in particular at the highest temperature (Fig 6b, S3 Table). During the algal decomposition, organic C in the overlying water and sediment interact with each other. Undegraded organic C in the overlying water deposits to the sediment and vise verse mineralization of stable organic C in sediments releases dissolved organic C to overlying water [22, 53]. Therefore, dynamics of DOC in the overlying water can be derived from both the algal decomposition and mineralization of sediment organic C. In the present study, DOC in the overlying water decreased at first at all temperatures due to the rapid mineralization of algal residues, then rise greatly due to the accelerated mineralization of organic C in sediments [54]. Although DOC in the overlying water varied in the similar pattern at all temperatures, there is an earlier reduction at the highest temperature, suggesting the acceleration of algal decomposition and DOC mineralization by warming [55]. Also, there was a greater rise in DOC in the overlying water at the later stage of algal decomposition at the highest temperature, indicating warming accelerated organic C mineralization in sediments [56]. This was further confirmed by the greater reduction in total organic C in sediments at the highest temperature (Fig 2f). Warming accelerated algal decomposition and mineralization of organic C in sediments were also observed in other lakes [18, 57].

Warming-induced changes in dynamics of DOC largely stimulated P release from sediments, mainly from the mineralization of sediment organic P in the present study. This was consistent with our third hypothesis, and was indicated by the synchronously occurred sharp rise of P release rate and reduction in sediment organic P at the early stage of algal decomposition at the highest temperature as compare with the corresponding increase in organic P at the lower temperatures (Fig 4c). Furthermore, total organic P in sediments was strongly and negatively correlated with DOC and SRP in water (S2 Table). In addition, reduction in organic P in sediments at the highest temperature was mainly in labile and moderate labile forms that extracted by NaHCO_3_ and NaOH solutions, as indicated by the dynamic and correlation analyses (S2 Fig). Interestingly and in contrast to 37°C, P release from sediments during algal decomposition at the lower temperatures was much smaller (near zero) and mainly from the mobilization of inorganic P in sediments. Algal decomposition greatly mobilized sediment inorganic P at all temperatures, as indicated by the great reduction (from 1186.33 mg Kg^−1^ to 918.71 mg Kg^−1^) in inorganic P (Fig 4b). Yet, the P released from the mobilization of inorganic P was mainly transformed to organic P instead of release to water at the lower temperatures, which led to the great accumulation of organic P in sediments (from 154.72 mg Kg^−1^ to 447.32 mg Kg^−1^) and slight increase in P release rate (Figs 3d and 4c). These interpretations were further confirmed by the negative correlation between P release rate and sediment inorganic P at the lowest temperature, in contrast to the negative correlation between P release rate and sediment organic P at the highest temperature (S3 Table).

Dissolved organic C is of great significance to the transformation of organic P in sediments [58–60]. Firstly, input of DOC had the “priming effect” on the mineralization of stable organic C and P in sediments [26, 27]. The easily-decomposable organic C serves as energy sources for microorganisms and is found to greatly promoted microbial biomass and phosphatase activity [61, 62]. Organic P is also an important C source for microorganisms under the C-limited conditions, the fracture of P ester bond (C-O-P) is an essential step for C mineralization [63]. Microbial demand for C was also found be an important driver of mineralization of organic P in sediments [64]. Predominant contribution of the reduction in organic P to the warming-induced reduction in sediment total P was also observed in previous simulation experiment [20]. DOC can also accelerate the release of phosphate adsorbed on the surfaces of Fe/Al oxides and minerals in sediments, as it is the strong competitor of phosphate ions for adsorption sites [65]. Since some low-molecular organic P are adsorbed on minerals, the desorption can not only release inorganic P adsorbed by minerals, but also contribute to the accelerated mineralization of organic P in sediments induced by input of dissolved organic C [66].

Reduction in Eh was another important way by which warming influenced P transformations and P release from sediments in the present study. The degradation of labile organic matter rapidly consumed oxygen, and decreased the Eh in water and sediments, which can be reinforced by warming [67]. This was true in the present study, as Eh in water decreased consistently with the decomposition of algal residues, and to a greater extent at 37°C than at lower temperatures (Fig 2b). In the anoxic and anaerobic system, Fe^3+^ is reduced to Fe^2+^, and phosphates originally combined with Fe^3+^ can be easily released from sediments [68, 69]. This probably be the main reason that labile inorganic P extracted by NH_4_Cl and NaHCO_3_ reduced synchronously with Eh [70]. Although pH is a key factor controlling both organic and inorganic P transformations [71], variation in pH may play a minor role in warming-induced P release from sediments in the present study, since sediment pH did not change with rising temperature and no significant correlations were observed between P release rate and pH in both water and sediments (Fig 2c and 2d, S3 Table). The pH increased significantly with rising temperature in the overlying water, but not in the sediment probably because of the high acid buffering capacity of sediments [72]. The same dynamic of the overlying water and sediment pH were also observed in many studies [73, 74].

### Non-linear responses of sediment P release rate to rising temperature

The most striking finding of this study was that the rate of P release from sediments did not change when incubated temperature rose from 20°C to 28°C, while greatly elevated when temperature continued to rise to 37°C (Fig 3d). Related physiochemical properties in water and sediments, such as Eh, pH and DOC, followed the similar patter with rising temperature. The breakdown of algal residues and mineralization of organic P depend on microorganisms [75]. So, the probable reason for this non-linear increase in P release from sediments is that growth and activities of microorganisms in the water and sediments respond to rising temperature in a non-linear pattern. It is found that relative growth of both soil bacterial and fungal communities almost did not change with rising temperature from 15°C to 30°C, while increased sharply and consistently from 30°C to 50°C [76, 77]. In a microcosm simulation experiment with the temperature rising from 5°C to 40°C, some C mineralization enzymes in sediments of a subtropical shallow freshwater lake also increased much greater when temperature rise from 30°C to 40°C than from 20°C to 30°C [56]. Soil P mineralization rate was also found to increase with temperature, to a greater extent when temperature exceeds 30°C [78]. Different sensitive of some microbial functional groups to rising temperature could also be partly responsible for the non-linear increase in P release from sediments [79, 80]. For example, phosphate supply in sediment pore water depended not only on the mineralization of organic P, but also on the presence or absence of electron acceptors and sulfate reducing bacteria [81, 82]. Temperature also affects these reactions related to P transformations. Clear mechanisms of this striking findings deserve further studies.

Beyond the next few decades, the magnitude of climate change depends primarily on cumulative emissions of greenhouse gases and aerosols and the sensitivity of the climate system to those emissions (high confidence). Projected changes range from 4.7°–8.6°F (2.6°–4.8°C) under the higher scenario (RCP8.5) to 0.5°–1.3°F (0.3°–1.7°C) under the much lower scenario (RCP2.6), for 2081–2100 relative to 1986–2005 (medium confidence) [1]. Furthermore, in recent years, the annual highest temperature exceeds 40°C in many regions. The temperature of water and sediments in lakes will increase accordingly, particularly in shallow lakes. Global lake surface temperature has been increasing between 1985 and 2009 (global mean = 0.34°C decade^−1^) [83]. Therefore, although the clear mechanisms underlying the nonlinear increase in sediment P release rate with rising temperature could not be fully explained in the present study, these findings at least suggest that further temperature increase may greatly accelerate P release rate from sediments to overlying water and thus aggravate algal blooms in eutrophic shallow lakes.

The present study was based on the laboratory simulative warming experiment using water and sediment collected from lake Chaohu. In our laboratory experiment, only water, sediment and algae are included, while external nutrient inputs and other components of the ecosystem were excluded. In the field conditions, external P loading can interact with P release from sediments, and other components of the ecosystem, such as zooplankton and fish, can also affect P transformation. Therefore, the main limitation of present study is the gap between the laboratory experiment and the field situation. However, considering that algae, sediments, and water are dominant pools of P in lakes, our results and findings are applicable in the field conditions to a large extent.

## Conclusions

In summary, this warming simulation experiment revealed that rising temperature greatly elevated endogenous P release from sediments during the decay of algal residues in a eutrophic shallow lake. More importantly, we found the mineralization of organic P in the sediment was the main source of P released to water under rising temperature, and warming-induced changes and dynamics of dissolved organic C played the dominant role in stimulating P release from sediments. Also interestingly, these great impacts of rising temperature on endogenous P release rate and related physiochemical occurred only when incubation temperature rise from 28°C to 37°C, but not from 21°C to 28°C, reflecting a non-linear temperature sensitivity of sediment P release rate under warming.

These findings greatly advanced our understanding of the relationships among algal blooms decomposition, climate warming, and lake eutrophication, and provide crucial information for decision-making in lake management under the background of global warming. Specifically, our findings highlight the dominant role dissolved organic C in stimulating P release from sediments, which suggests the positive feedbacks between warming, algal decomposition and sediment P release. Warming can facilitate the growth of algae, and then the decomposition processes of algae residues can stimulate sediment P release, as well as aggravate warming due to increased CO_2_ emission. Therefore, Due to these, strategies are imperative to control algal growth, dissolved organic C, and sediment P release in eutrophic lakes under the background of global warming.

## Supporting information

S1 Table Results of one-way ANOVA (*p <* 0.05) of the effects of incubation temperature on water and sediment variables at the same sampling date. (PDF)

S2 Table Pearson’s correlation coefficients between sediment P fractions and related physicochemical characteristics of water and sediments. (PDF)

S3 Table Pearson’s correlation coefficients between sediment P release flux and physicochemical properties of water and sediments at different incubation temperatures. (PDF)

S1 Fig Sediment P fractions in different temperature. * and ** indicate significant effect at *p* < 0.05 and *p* < 0.01, respectively.
(DOCX)

S2 Fig Pearson’s correlation coefficients between sediment P fractions and related water biochemical properties (a–c), sediment P fractions and related sediment biochemical properties (d–f) and sediment P fractions (g–i). EC: electrical conductivity; Eh: redox potential; DOC: dissolved organic C; TOC: total organic C; AlPase: alkaline phosphatase activity; TP: total P in water; TDP: dissolved total P; SRP: soluble reactive P; Pi: total inorganic P; Po: total organic P; Pt: total.
(DOCX)
