# Supplementary material for: Warming accelerated phosphorus release from the sediment of Lake Chaohu during the decomposition of algal residues: A simulative study
Source: PLoS One. 2025 Jan 15;20(1):e0314534. doi: 10.1371/journal.pone.0314534 (PMC11734940; doi:10.1371/journal.pone.0314534)
Supplement: S1 Table — (PDF) [file pone.0314534.s001.pdf]

Table S1. Results of one-way ANOVA (p < 0.05) of the effects of incubation temperature on water and sediment variables at the same sampling date

variables	Incubation time（days）					
	1d	5d	9d	13d	17d	22d
Water						
pH	0.058*	0.027**	0.044*	0.066*	0.027*	0.191
Electrical conductivity	0.027*	0.027*	0.027*	0.027*	0.027*	0.024*
Oxidation-reduction potential	0.061*	0.024*	0.051*	0.066*	0.027*	0.051*
Dissolved organic C	0.061*	0.587	0.027*	0.051*	0.039*	0.066*
Alkaline phosphatase activity	0.027*	0.430	0.058*	0.301	0.561	0.027*
Total P	0.055*	0.027*	0.059*	0.051*	0.066*	0.027*
Total dissolved P	0.050*	0.027*	0.066*	0.051*	0.027*	0.027*
Soluble reactive P	0.099*	0.051*	0.059*	0.051*	0.027*	0.027*
Sediment						
pH	0.646	0.066*	0.067*	0.790	0.670	0.288
Total organic C	0.039*	0.027*	0.051*	0.733	0.061*	0.099
Alkaline phosphatase activity	0.051*	0.202	0.051*	0.051*	0.837	0.066*
Total P	0.000**	0.000**	0.001**	0.000**	0.094	0.125
Inorganic P	0.066*	0.099	0.329	0.061*	0.027*	0.148
Organic P	0.027*	0.027*	0.148	0.288	0.252	0.061*
Proportion of organic P	0.003**	0.013*	0.154	0.290	0.159	0.036*
NH_4Cl-Pi	0.126	0.132	0.002**	0.046*	0.947	0.527
$NaHCO_3$-Pi	0.003**	0.349	0.074	0.043*	0.001**	0.037*
NaOH-Pi	0.587	0.000**	0.013*	0.001**	0.150	0.001**
HCl-Pi	0.625	0.124	0.081	0.000**	0.027*	0.010*
NH_4Cl-Po	0.064*	0.123	0.304	0.010*	0.060*	0.066*
$NaHCO_3$-Po	0.001**	0.368	0.002**	0.787	0.000**	0.000**
NaOH-Po	0.004**	0.001**	0.005**	0.807	0.301	0.002**
HCl-Po	0.014*	0.002**	0.193	0.002**	0.000**	0.001**

*, ** represent the significant differences at the 0.05 and 0.01 levels, respectively.
