# Supplementary material for: Warming accelerated phosphorus release from the sediment of Lake Chaohu during the decomposition of algal residues: A simulative study
Source: PLoS One. 2025 Jan 15;20(1):e0314534. doi: 10.1371/journal.pone.0314534 (PMC11734940; doi:10.1371/journal.pone.0314534)
Supplement: S2 Table — (PDF) [file pone.0314534.s002.pdf]

Table S2. Pearson's correlation coefficients between sediment P fractions and related physicochemical characteristics of water and sediments.

	NH_4Cl-P_i	$NaHCO_3$-P_i	NaOH-P_i	HCl-P_i	NH_4Cl-P_o	$NaHCO_3$-Po	NaOH-P_o	HCl-P_o	pHw	EC	Eh	DOC	$AlPase_W$	TP	TDP	SRP	pHs	TOC	$AlPase_S$	Pt	Pi	Po
NH4Cl-Pi	**1**																					
NaHCO3-Pi	**0.762****	**1**																				
NaOH-Pi	**0.094**	**-0.048**	**1**																			
HCl-Pi	**-0.134**	**-0.255**	**-0.176**	**1**																		
NH4Cl-Po	**-0.194**	**0.110**	**-0.124**	**-0.019**	**1**																	
NaHCO3-Po	**0.304***	**0.247**	**-0.065**	**0.040**	**0.039**	**1**																
NaOH- Po	**-0.571****	**-0.456****	**0.030**	**0.174**	**-0.181**	**0.100**	**1**															
HCl-Po	**-0.126**	**-0.299****	**0.070**	**-0.035**	**-0.211**	**-0.236**	**0.326***	**1**														
pHw	**-0.119**	**-0.272***	**0.151**	**-0.143**	**-0.292***	**0.031**	**0.110**	**0.043**	**1**													
EC	**-0.587****	**-0.832****	**0.107**	**0.080**	**-0.250**	**-0.304***	**0.311***	**0.119**	**0.603****	**1**												
Eh	**0.474****	**0.680****	**-0.125**	**0.034**	**0.182**	**0.454****	**-0.144**	**0.101**	**-0.483****	**-0.821****	**1**											
DOC	**-0.182**	**-0.291***	**0.050**	**0.125**	**-0.166**	**0.358****	**0.446****	**0.100**	**0.629****	**0.397****	**-0.073**	**1**										
$AlPase_W$	**0.198**	**0.294***	**-0.169**	**0.286***	**0.152**	**0.431****	**-0.033**	**-0.520****	**0.074**	**-0.176**	**0.291***	**0.261**	**1**									
TP	**-0.174**	**-0.291***	**0.230**	**-0.197**	**0.019**	**-0.403****	**-0.217**	**-0.291***	**0.372****	**0.604****	**-0.610****	**0.064**	**-0.036**	**1**								
TDP	**-0.098**	**-0.217**	**0.206**	**-0.201**	**0.032**	**-0.312***	**-0.255**	**-0.343***	**0.418****	**0.571****	**-0.578****	**0.094**	**0.016**	**0.973****	**1**							
SRP	**-0.154**	**-0.298***	**0.226**	**-0.142**	**0.086**	**-0.300***	**-0.226**	**-0.207**	**0.463****	**0.618****	**-0.596****	**0.132**	**0.007**	**0.932****	**0.957****	**1**						
pHs	**-0.720****	**-0.788****	**-0.122**	**0.126**	**-0.057**	**-0.179**	**0.445****	**0.257**	**0.396****	**0.703****	**-0.548****	**0.417****	**-0.174**	**0.126**	**0.066**	**0.142**	**1**					
TOC	**-0.192**	**-0.265**	**0.081**	**0.074**	**-0.079**	**-0.035**	**0.296***	**-0.053**	**-0.128**	**0.134**	**-0.058**	**0.033**	**0.118**	**0.142**	**0.109**	**0.034**	**0.107**	**1**				
$AlPase_S$	**0467****	**0.624****	**-0.089**	**-0.071**	**0.120**	**0.207**	**-0.162**	**-0.030**	**-0.318***	**-0.632****	**0.750****	**-0.158**	**0.237**	**-0.419****	**-0.396****	**-0.370****	**-0.425****	**-0.240**	**1**			
Pt	**0.467****	**0.302***	**0.442****	**-0.306***	**-0.087**	**0.148**	**-0.142**	**0.205**	**0.089**	**-0.074**	**0.139**	**-0.293***	**0.094**	**0.096**	**0.149**	**0.133**	**-0.507****	**0.141**	**0.116**	**1**		
Pi	**-0.007**	**0.028**	**0.315***	**-0.048**	**0.005**	**0.288***	**0.214**	**0.118**	**0.388***	**0.150**	**0.024**	**0.448****	**0.271***	**0.183**	**0.238**	**0.335***	**0.069**	**0.087**	**0.173**	**0.330***	**1**	
Po	**0.251**	**0.246**	**0.185**	**0.042**	**-0.109**	**-0.026**	**-0.215**	**-0.076**	**-0.148**	**-0.351****	**0.245**	**-0.284***	**0.008**	**-0.251**	**-0.255**	**-0.369****	**-0.299***	**0.075**	**0.018**	**0.495****	**-0.429****	**1**

pHw: water pH; pHs: sediment pH; EC: electrical conductivity; Eh: redox potential; DOC: dissolved organic C; TOC: total organic C; $AlPase_W$: water alkaline phosphatase activity; $AlPase_S$: sediment alkaline phosphatase activity; TP: total P in water; TDP: dissolved total P; SRP: soluble reactive P; Pi: total inorganic P; Po: total organic P; Pt: total P.
