# Supplementary material for: Warming accelerated phosphorus release from the sediment of Lake Chaohu during the decomposition of algal residues: A simulative study
Source: PLoS One. 2025 Jan 15;20(1):e0314534. doi: 10.1371/journal.pone.0314534 (PMC11734940; doi:10.1371/journal.pone.0314534)
Supplement: S3 Table — (PDF) [file pone.0314534.s003.pdf]

Table S3. Pearson's correlation coefficients between sediment P release flux and physicochemical properties of water and sediments at different incubation temperatures.

Temperature (°C)	Overlying water							Sediments					
	pH	Eh	DOC	AlPase	Total P	TDP	SRP	pH	TOC	AlPase	Total P	Inorganic P	Organic P
21	-0.154	-0.832**	-0.408*	-0.001	0.420	0.338	-0.026	0.135	0.525*	-0.780**	-0.222	-0.833**	0.486*
28	-0.405	-0.877**	-0.257**	-0.273	0.680**	0.706**	0.660**	0.270	0.909**	-0.780**	-0.123	-0.448	-0.176
37	0.004	-0.753**	-0.671**	-0.017	0.564*	0.801**	0.825**	0.167	-0.331	-0.456	-0.442	-0.282	-0.555*

* and ** represent the significant differences at $p = 0.01$ and $p = 0.05$, respectively. Eh: redox potential; DOC: dissolved organic C; AlPase: alkaline phosphatase activity; TDP: dissolved total P; SRP: soluble reactive P; TOC: total organic C.
