# Supplementary material for: Warming accelerated phosphorus release from the sediment of Lake Chaohu during the decomposition of algal residues: A simulative study
Source: PLoS One. 2025 Jan 15;20(1):e0314534. doi: 10.1371/journal.pone.0314534 (PMC11734940; doi:10.1371/journal.pone.0314534)
Supplement: S1 Fig — * and ** indicate significant effect at p < 0.05 and p < 0.01, respectively. (DOCX) [file pone.0314534.s004.docx]

**
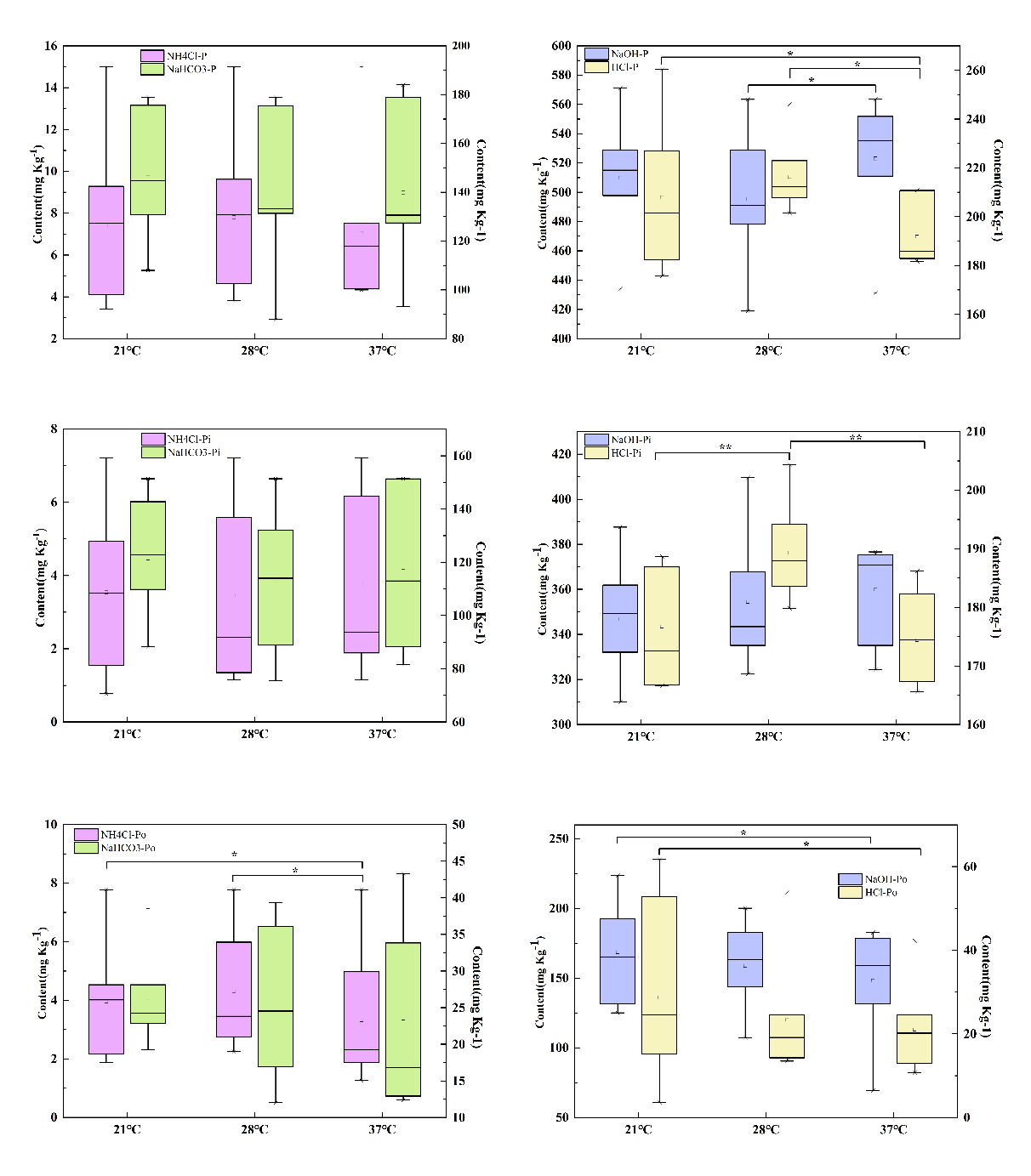
**

**Fig S1. Sediment P fractions in different temperature.** ^*^and ^**^  indicate significant effect at *p* ﹤ 0.05 and  *p* ﹤ 0.01, respectively.
