# Supplementary material for: Warming accelerated phosphorus release from the sediment of Lake Chaohu during the decomposition of algal residues: A simulative study
Source: PLoS One. 2025 Jan 15;20(1):e0314534. doi: 10.1371/journal.pone.0314534 (PMC11734940; doi:10.1371/journal.pone.0314534)
Supplement: S2 Fig — EC: electrical conductivity; Eh: redox potential; DOC: dissolved organic C; TOC: total organic C; AlPase: alkaline phosphatase activity; TP: total P in water; TDP: dissolved total P; SRP: soluble reactive P; Pi: total inorganic P; Po: total organic P; Pt: total. (DOCX) [file pone.0314534.s005.docx]

**
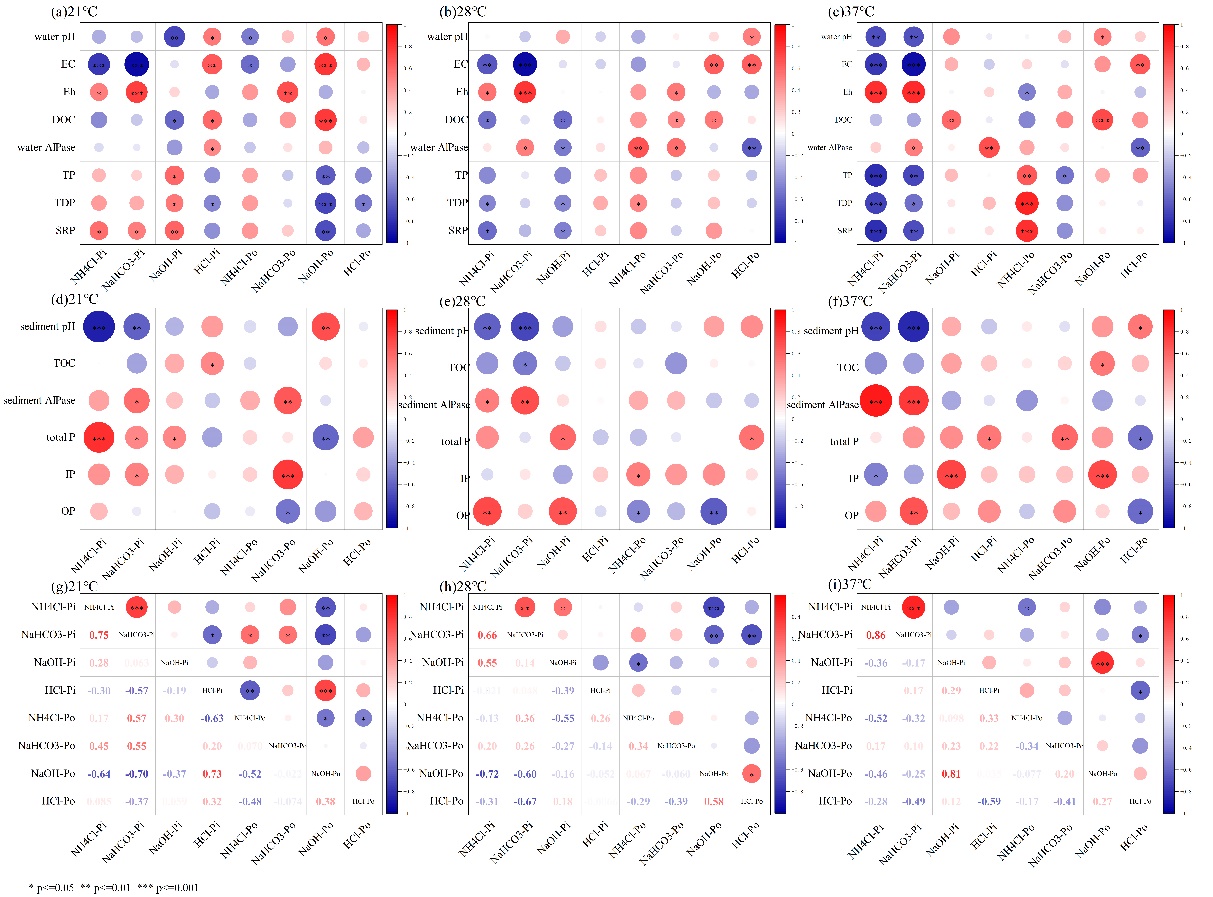
**

**Fig S2. Pearson's correlation coefficients between sediment P fractions and** **related water biochemical properties (a–c), sediment P fractions and related sediment biochemical properties (d–f) and sediment P fractions (g–i).** EC: electrical conductivity; Eh: redox potential; DOC: dissolved organic C; TOC: total organic C; AlPase: alkaline phosphatase activity; TP: total P in water; TDP: dissolved total P; SRP: soluble reactive P; Pi: total inorganic P; Po: total organic P; Pt: total.
